# Maxillary reconstruction using rectus femoris muscle flap and sagittal mandibular ramus/coronoid process graft pedicled with temporalis muscle

**DOI:** 10.4317/medoral.22505

**Published:** 2018-09-28

**Authors:** Weihong Wang, Biao Xu, Jin Zhu, Chun Yang, Shiying Shen, Yemei Qian

**Affiliations:** 1 M.D, Associate professor, Department of Oral and Maxillofacial Surgery, Affiliated Stomatology Hospital of Kunming Medical University. Kunming 650106, China; 2 M.D, Ph.D, Professor, Department of Oral and Maxillofacial Surgery, Affiliated Stomatology Hospital of Kunming Medical University. Kunming 650106, China; 3 M.D, Associate professor. Department of Oral Anatomy and Pathology of Kunming Medical University, Kunming 650101, China

## Abstract

**Background:**

Maxillary reconstruction using various pedicled and free-tissue transfer techniques with bone graft or without bone graft has some drawbacks. In this study, we demonstrate maxillary reconstruction using femoris rectus muscle flap and sagittal mandibular ramus/coronoid process graft pedicled with temporalis muscle through the modified lateral lip-submandibular approach.

**Material and Methods:**

Nine patients suffering from maxillary defects secondary to maxillary cancer ablation, who underwent maxillary reconstruction using rectus femoris muscle flap and sagittal mandibular ramus/coronoid process graft pedicled with temporalis muscle, were enrolled into this study between November 2015 and August 2017.

**Results:**

All patients who underwent the maxillary reconstruction using femoris rectus muscle flap and sagittal mandibular ramus/coronoid process graft pedicled with temporalis muscle presented satisfactory postoperative function, with adequate mouth opening, optimal esthetic outcome and no restrictions on the diet. Every rectus femoris muscle flaps mucosalized well within five weeks. No donor site functional impairment or complications were observed.

**Conclusions:**

The technique is a feasible and acceptable technique for the maxillary reconstructions. It is safe, quick and simple to harvest. It also presents an optimal esthetic and satisfactory functional outcome with the advantage of low morbidity of the donor site. Combined with the three-dimension reconstruction, this technique can improve the postoperative outcomes.

** Key words:**Rectus femoris muscle flap, sagittal mandibular ramus, coronoid process graft.

## Introduction

The maxilla is the importance of the central portion of the face, which provides much of the facial appearance, as well as supports speech and communication, and the function of swallowing and chewing. The maxillary defects caused by tumor resection often result in severe function and cosmetic deformity. In recent years, with the development of microsurgical technology, maxillary reconstruction using various pedicled and free-tissue transfer techniques with bone graft or without bone graft such as free vascularized fibular flap (1,2) and free rectus abdominis flap with bone grafts (3,4), can be a good recovery of the patient’s facial shape and mouth and jaw function. However, some drawbacks cannot be overlooked. For instance, the fibular flap often led to delayed mobilization of the leg due to numbness, toe contracture and abnormal ambulatory movement (5), thereby some patients with maxillary carcinoma, who came from a countryside, did not agree in going through the surgical procedure, especially those living mountainous areas. The free rectus abdominis flap with bone grafts needs higher skill to harvest, which may result in complications such as abdominal incision hernias (3,6). Here, we describe the technique and evaluate the reliability of the maxillary reconstruction in patients with malignant tumor using rectus femoris muscle flap combined with sagittal mandibular ramus/coronoid process graft pedicled with temporalis muscle. This surgical technique improved the appearance and oral function and enhanced the quality of life of all patients.

## Material and Methods

Consecutive eight female and one male patients with maxillary carcinoma were admitted to the Department of Oral and Maxillofacial Surgery, Affiliated Stomatology Hospital of Kunming Medical University between November 2015 and August 2017. This research was approved by the ethics committee of Kunming Medical University.

The pathological diagnosis and the clinical information were collected. The average age of these patients at the time of the admission to the hospital were 47.8 years old, ranging from 30 to 58 years old. Five patients presented maxillary mucoepidermoid carcinoma and three presented adenoid cystic carcinoma and one with squamous cell carcinoma. All cases were classified from T2N0M0 to T4N1M0 (TNM classification). They were from Class II defect (without involving the orbit) and Class III defect (involving the orbital adnexa with orbital retention) according to the new classification for the maxillectomy defect (7) and NCCN guidelines (8). Clinical data of the nine patients are summarized in  Table 1. Maxillary reconstruction was performed using rectus femoris muscle flap combined with sagittal mandibular ramus/coronoid process graft pedicled with temporalis muscle.

**Table 1:**
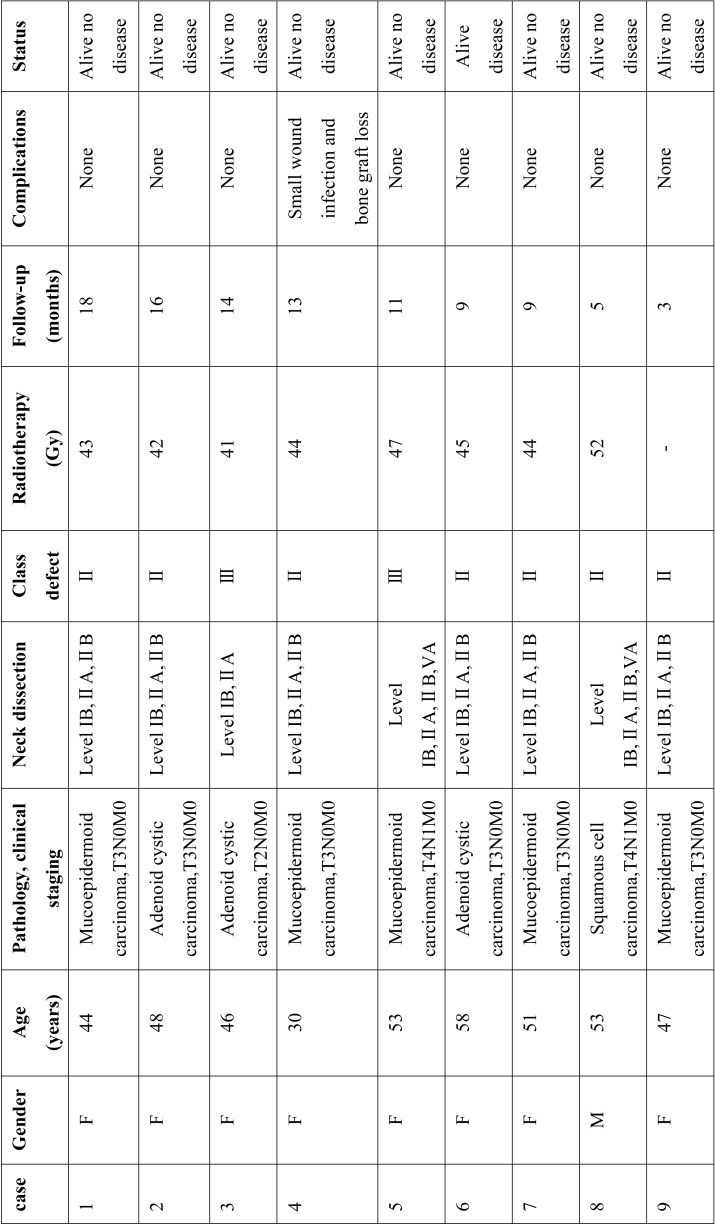
Demographic and clinical characteristics of nine patients with maxillary malignant tumors.

**Figure 1:**
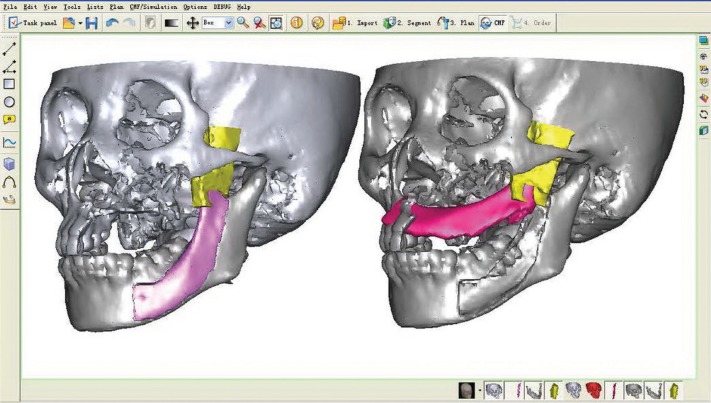
Three-dimensional virtual operation in reconstruction of maxilllary defect by sagittal mandibular ramus/coronoid process graft.

**Figure 2:**
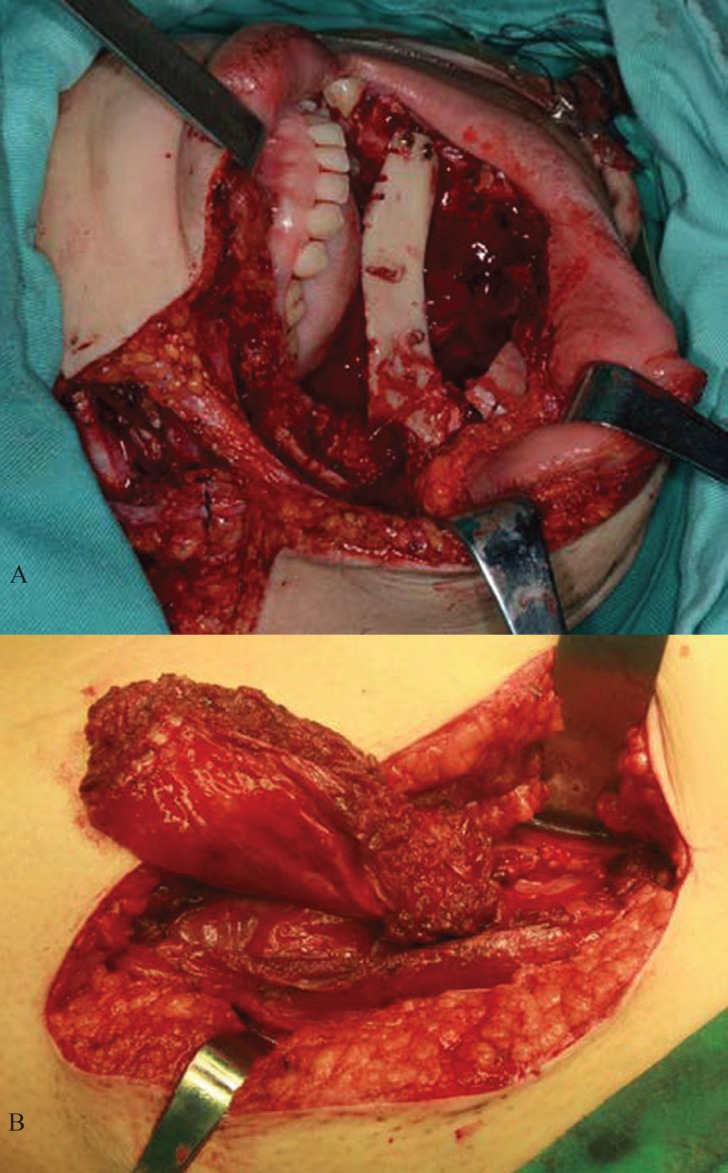
A. The sagittal mandibular ramus/coronoid process pedicled with temporalis muscle was fixed to the maxillary residual with titanium screws. B. The rectus femoris muscle flap was harvested.

According to the DICOM data obtained from the scanned CT, the tumour resection and maxillary reconstruction were simulated using the software program SimPlantTM (version 11.04, Materialise NV, Leuven, Belgium) (Fig. 1). The modified lateral lip-submandibular approach was performed following the elective neck dissection marked, and the sagittal mandibular ramus/coronoid process graft pedicled on the temporalis muscle was harvested as described in our previous study (9) (Fig. 2). Simultaneously, an anteromedial incision along the lateral circumflex femoral artery (LCFA) was made to harvest a rectus femoris muscle flap from the donor thigh by another surgical team. The dissection was made down to the level of the rectus femoris muscle fascia. The medial branch of the descending LCFA along the branch of the descending LCFA was dissected downward deep to the rectus femoris muscle, in which the vascular pedicle contained the partial rectus femoris muscle component. Then the partial rectus femoris muscle flap with the surface fascia was harvested using an ultrasonic-harmonic scalpel according to the size of the maxillary defect (Fig. 2). Finally, the pedicle was gently transferred to the recipient site through the tunnel between the oral floor and the mandibular medial surface after meticulous haemostasis, and then the anastomosis of the artery and the vein were performed. The muscle and fascia of the flap covered the intraoral lining of the bone flap, and were sutured to the maxillary mucosa. After the surgery, a suction drainage was placed and the ipsilateral rotation of the patient’s head was recommended for 5 days. Subsequently, extensive physiotherapy exercise for the temporomandibular joint was recommended, and adjuvant radiotherapy was administered at a mean dose of 45 Gy.

## Results

**Figure 3:**
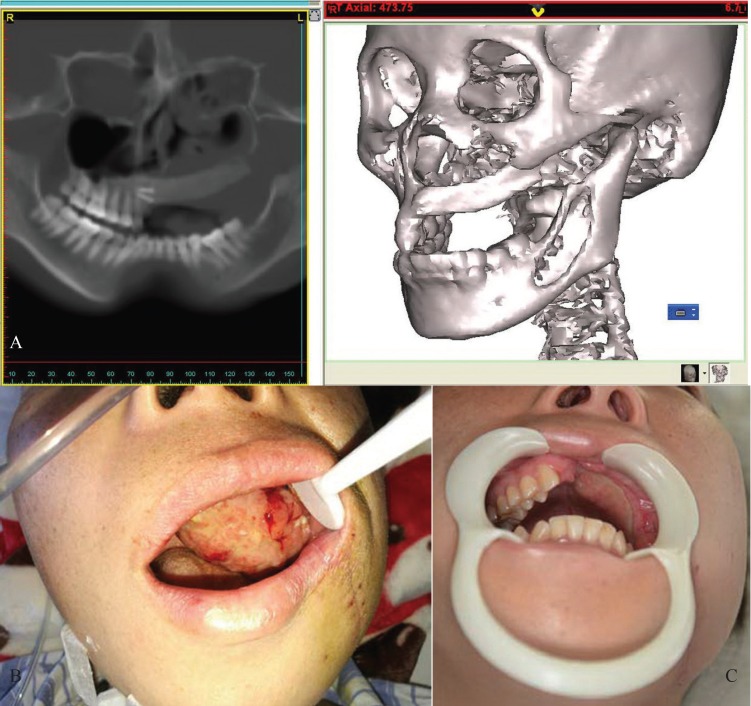
A. Three-dimensional computed tomography scan confirmed volume maintenance and anatomic continuity of the graft. B. The frontal view two weeks later. C. Six months later.

In all patients, the surgical procedure was deemed successful, all rectus femoris muscle flaps mucosalized well within five weeks, only there was a wound infection at the one patient’s cheek skin but eventually healed well following drainage. The patients were followed up during different periods varying between from 3 to 18 months (mean 11 months). Acceptable maxillomandibular relationship, satisfactory esthetic results with good facial contour and symmetrical lower lip symmetry were achieved based on clinical examination (Fig. 3). In addition, all patients returned to an unrestricted diet since mouth opening was more than 2.5 cm. Wound healing in the donor site morbidity and other complications were not observed such as diplopia, ectropion and loss of both knee extension and quadriceps strength. Postoperative computed tomography (CT) scan showed normal orbit morphology and ossification of the transplanted bone grafts (Fig. 3). Unfortunately, one sagittal mandibular ramus/coronoid process graft had to be removed in the patient with the wound infection even eventually healed well following drainage because it exposed after the postoperative radiotherapy four months later (eight months after the operation). One patient developed local recurrence in the maxilla. That patient refused further treatment during the period of follow-up.

## Discussion

In previous studies about maxillary reconstruction, diverse methods, such as reverse facial-submental artery island flap (10,11), radial forearm free flap(12), free vascularized iliac crest flap (13) and anterolateral thigh(ALT) flap have been reported (14,15). However, submental island flap is strongly not recommended for patients with clinically cervical lymph node positive due to the possibility of metastatic tissue transfer and recurrence in the flap base (16). Radial forearm free flap has some disadvantages as it presented esthetic problems and morbidity at the donor site and postoperative shrinkage (17). Vascularized iliac crest flap present delayed healing and breakdown of the suture line at the iliac site (6). ALT flap technique also showed some limitations. The anatomic variations of this surgical area, including complete absence of identifiable perforators and bulky oral component, especially female patients, which thereby make it not the best-practice (18,19). In this present study, the thickness of subcutaneous fat was nearly up to 50 millimeters in one of patient’s thigh (Fig. 2). Therefore, to avoid these problems, we used rectus femoris muscle flap and sagittal mandibualar ramus/coronoid process graft pedicled with temporalis muscle for maxillary reconstruction of Class II and III defect.

In this study, the sagittal mandibular ramus/coronoid process graft pedicled with temporalis muscle showed to be a simple and reliable technique, similar to the orbital floor reconstruction with a coronoid temporalis pedicle flap (20-22). It also recommended for the reconstruction of the anterior maxillary bone defect. While the rectus femoris muscle flap was used to repair the palatal mucosa. In fact, the interiorly distal segment of the bone flap is exposed, the risk of infection and subsequent removal may occur. Thus, we used the partial muscle and fascia of the flap to wrap up the bone flap at nasal aspect before it was sutured to the maxillary mucosa, this may obviate the risk of extrusion or exposure, minimizes the risk of infection. And reduce the impact of postoperative irradiation. Clinically, the thickness of the rectus femoris muscle flap can be easily adjusted to fill the maxillary defect or including the orbital defect, this advantage attributes to its anatomic characteristics. Anatomically, the blood supply of rectus femoris muscle flap and anteromedial thigh (AMT) flap based on the same artery, known as the medial branch of the descending LCFA, it shares the same grand vessel pedicle with the lateral branch of the descending LCFA which supplies the vastus lateralis. Both the medial and the lateral branch own the common vessel, namely the LCFA. The former runs through the intermuscular space between the vastus medialis and the rectus femoris, and then constantly into the rectus femoris, while the latter always runs through the intermuscular space between the vastus lateralis with variant perforator (23,24). Therefore, rectus femoris flap is more feasible than adipofascial ALT flap as far as the blood supply is concerned. In this series of cases, there were no complications in the donor leg such as loss of both knee extension force and quadriceps strength, which was consistent with the previous studies even though the total rectus femoris muscle was used in the literature (25). Of course, the vessel length of the former is not enough as well as the diameter compared with the latter if the harvested vascular pedicle excluded the partial rectus femoris muscle component. On the contrary, the mean length of the vascular pedicle and the diameter is appropriate if the partial rectus femoris muscle component includes. Thus, both the length and the diameter of the vessel are enough anastomosed with the facial vessels. Fortunately, the modified lateral lip-submandibular approach in our study had advantages of wide exposure and that the recipient and supplying vessels can be easily adjusted and anastomosed at the same operative field as described in our previous study (9). In addition, coronoidectomy could reduce the likelihood of trismus following maxillectomy especially for those who had adjuvant radiotherapy. In this case, adjuvant physiotherapy exercises early for mouth opening are necessary (26-28). In this study, no trismus occurred in this series even after postoperative radiotherapy at the period of follow-up.

## Conclusions

In summary, the rectus femoris muscle flap combined with sagittal mandibular ramus/coronoid process graft showed to be a feasible and acceptable technique for maxillary reconstructions following the maxillary carcinoma ablation. It is safe, and simple to harvest reliable. Moreover, it can provide satisfactory result and minimal donor site morbidity is observed. This technique flaps, however, cannot not provide essential bulk of bone for implant placement. Therefore, if the postoperative implant is suitable, other techniques including osseocutaneous free tissue should be considered such as free vascularized iliac crest flap and free vascularized fibular flap.

## References

[1] Wang YY, Fan S, Zhang HQ, Lin ZY, Ye JT, Li JS (2016). Virtual Surgical Planning in Precise Maxillary Reconstruction With Vascularized Fibular Graft After Tumor Ablation. J Oral Maxillofac Surg.

[2] Shen Y, Li J, Ow A, Wang L, Lv MM, Sun J (2017). Acceptable clinical outcomes and recommended reconstructive strategies for secondary maxillary reconstruction with vascularized fibula osteomyocutaneous flap: A retrospective analysis. J Plast Reconstr Aesthet Surg.

[3] Sekido M, Yamamoto Y, Makino S (2006). Maxillary reconstruction using a free deep inferior epigastric perforator (DIEP) flap combined with vascularised costal cartilages. J Plast Reconstr Aesthet Surg.

[4] Bianchi B, Bertolini F, Ferrari S, Sesenna E (2006). Maxillary reconstruction using rectus abdominis free flap and bone grafts. Br J Oral Maxillofac Surg.

[5] Li P, Fang Q, Qi J, Luo R, Sun C (2015). Risk Factors for Early and Late Donor-Site Morbidity After Free Fibula Flap Harvest. J Oral Maxillofac Surg.

[6] Ling XF, Peng X, Samman N (2013). Donor-site morbidity of free fibula and DCIA flaps. J Oral Maxillofac Surg.

[7] Brown JS, Shaw RJ (2010). Reconstruction of the maxilla and midface: introducing a new classification. Lancet Oncol.

[8] (2016). National Comprehensive Cancer Network. Head and Neck Cancers, version 1. https://www.nccn.org/professionals/physician_gls/PDF/head-and-neck.pdf.

[9] Wang WH, Xu B (2013). Maxillary reconstruction using vascularized fibular osteomyocutaneous flap and iliac bone through modified lateral lip-submandibular approach. J Craniofac Surg.

[10] Chow TL, Fung SC, Choi CY, Ho LI, Kwan WWY (2016). Maxillary reconstruction with pedicled reverse-flow submental osteocutaneous mandible chimeric flap. J Oral Maxillofac Surg Med Pathol.

[11] Chen WL, Zhou M, Ye JT, Yang ZH, Zhang DM (2011). Maxillary functional reconstruction using a reverse facial artery-submental artery mandibular osteomuscular flap with dental implants. J Oral Maxillofac Surg.

[12] Costa H, Zenha H, Sequeira H, Coelho G, Gomes N, Pinto C (2015). Microsurgical reconstruction of the maxilla: Algorithm and concepts. J Plast Reconstr Aesthet Surg.

[13] Grinsell D, Catto-Smith HE (2015). Modifications of the deep circumflex iliac artery free flap for reconstruction of the maxilla. J Plast Reconstr Aesthet Surg.

[14] Bianchi B, Ferri A, Ferrari S, Copelli C, Sesenna E (2009). Maxillary reconstruction using anterolateral thigh flap and bone grafts. Microsurgery.

[15] Kekatpure VD, Hedne N, Chavre S, Pillai V, Trivedi N, Kuriakose MA (2014). Versatility of adipofascial anterolateral thigh flap for reconstruction of maxillary defects with infratemporal fossa extension. Craniomaxillofac Trauma Reconstr.

[16] Rahpeyma A, Khajehahmadi S (2014). Submental artery island flap in intraoral reconstruction: a review. J Craniomaxillofac Surg.

[17] Villaret DB, Futran NA (2003). The indications and outcomes in the use of osteocutaneous radial forearm free flap. Head Neck.

[18] Lakhiani C, Lee MR, Saint-Cyr M (2012). Vascular anatomy of the anterolateral thigh flap: a systematic review. Plast Reconstr Surg.

[19] De Beule T, Van Deun W, Vranckx J, de Dobbelaere B, Maleux G, Heye S (2016). Anatomical variations and pre-operative imaging technique concerning the anterolateral thigh flap: guiding the surgeon. Br J Radio.

[20] Curioni C, Toscano P, Fioretti C, Salerno G (1983). Reconstruction of the orbital floor with the muscle-bone flap (temporal muscle with coronoid process). J Maxillofac Surg.

[21] Pryor SG, Moore EJ, Kasperbauer JL, Hayden RE, Strome SE (2004). Coronoid-temporalis pedicled rotation flap for orbital floor reconstruction of the total maxillectomy defect. Laryngoscope.

[22] Yu M, Qin XJ, Zhang CP, Xu LQ (2016). A modified technique for reconstruction of a total maxillary defect. Br J Oral Maxillofac Surg.

[23] Agostini T, Lazzeri D, Spinelli G (2014). Anterolateral thigh flap thinning: techniques and complications. Ann Plast Surg.

[24] Wang WH, Deng JY, Xu B, Zhu J, Xia B, Zhang BJ (2014). Double anterior (anterolateral and anteromedial) thigh flap for oral perforated defect reconstruction. J Craniomaxillofac Surg.

[25] Daigeler A, Dodic T, Awiszus F, Schneider W, Fansa H (2005). Donor-site morbidity of the pedicled rectus femoris muscle flap. Plast Reconstr Surg.

[26] Talmi YP, Horowitz Z, Yahalom R, Bedrin L (2004). Coronoidectomy in maxillary swing for reducing the incidence and severity of trismus-a reminder. J Craniomaxillofac Surg.

[27] Ren WH, Ao HW, Lin Q, Xu ZG, Zhang B (2013). Efficacy of mouth opening exercises in treating trismus after maxillectomy. Chinese medical journal.

[28] Yanamoto S, Yamada S, Takahashi H, Naruse T, Shigeta T, Minamikawa T (2014). Benefits of maxillectomy with internal dissection of the masticator space by transmandibular approach in the surgical management of malignant tumours of the upper gingiva and hard palate: a clinical review of 10 cases. Int J Oral Maxillofac Surg.

